# No-Show Rates in an Academic Otolaryngology Practice Before and During the COVID-19 Pandemic

**DOI:** 10.7759/cureus.54015

**Published:** 2024-02-11

**Authors:** Brian T Yuhan, Mayuri A Yasuda, Radhika Joshi, Steven Charous, Agnes Hurtuk

**Affiliations:** 1 Otolaryngology - Head and Neck Surgery, Loyola University Medical Center, Maywood, USA

**Keywords:** otolaryngology, ambulatory, nonattendance, no-show, outpatient clinic

## Abstract

Objective: Our objectives were to determine the no-show and nonattendance rate for an outpatient academic otolaryngology practice, to identify patient and systemic factors associated with nonattendance, and to evaluate the impact that the COVID-19 pandemic had on the rate of nonattendance.

Methods: This is a retrospective review of the Epic practice management and billing reports from all scheduled outpatient visits at a multi-physician, academic, general, and sub-specialty otolaryngology practice from January 2019 to December 2021.

Results: Over three years, 121,347 clinic visits were scheduled in the otolaryngology practice. The overall nonattendance rate was 18.3%. A statistically significant increase in nonattendance was noted during the COVID-19 pandemic (16.8% vs. 19.8%, p < 0.001). The rate of nonattendance in patients of younger age (under 18 years) (p <0.001), female gender (p=0.03), afternoon appointments (p=0.04), and extended time between the day of scheduling and the day of appointment (p <0.001) increased. Head and neck clinics were found to have the lowest nonattendance rates, while pediatric otolaryngology clinics had the highest (12.6% vs. 21.3%). On multivariate regression, younger age (p < 0.001), female gender (p=0.01), afternoon appointments (p< 0.001), and online self-scheduling (p< 0.001) were significantly associated with nonattendance.

Conclusions: Both patient and appointment-related factors were found to impact rates of nonattendance in this academic otolaryngology practice. In this study, young age, female gender, afternoon appointments, and online self-scheduling were associated with increased nonattendance. In addition, the COVID-19 pandemic significantly impacted no-show rates across all otolaryngologic subspecialties.

## Introduction

Nonattendance, or missed scheduled visits without any notification to the clinic, is a common issue that disrupts modern medical practice. For the patient, nonattendance may detrimentally impact that individual’s health, leading to poorer health-related outcomes, as well as affecting another patient’s ability to be expeditiously scheduled, evaluated, and treated. This leads to longer wait times for scheduling clinic visits, decreased patient satisfaction, and increased reliance on urgent care or emergency department visits [1]. For the practice, missed appointments lead to loss of clinical time, personnel, and utility overhead, increased stress, and decreased productivity at a time when productivity is paramount. Missed visits have a significant financial impact on entire healthcare systems by reducing the efficiency of resource utilization. One study from the University of California, San Francisco (UCSF) estimated approximately 67,000 no-show visits annually from the UCSF system alone at a cost of seven million dollars annually [2].

Multiple factors have been shown to impact clinic nonattendance, including the lack of transportation, impact of missed work, age, gender, insurance, and other financial barriers [3-5]. This initial evaluation of nonattendance and its effect on clinical practice is timely, as the COVID-19 pandemic has highlighted various topics regarding healthcare inequality, equitability, and access to care. To the best of our knowledge, this would be the first study evaluating the impact that the COVID-19 pandemic had on the rate of nonattendance for an academic otolaryngology practice. This article was previously presented as a meeting abstract and poster at the American Academy of Otolaryngology - Head and Neck Surgery (AAO-HNSF) Annual Meeting on September 11th, 2022. This study also aims to identify patient and system factors associated with nonattendance with the hope that this data may be used to identify strategies to positively influence clinic appointment adherence and improve health resource utilization.

## Materials and methods

An institutional review board (IRB) exemption was obtained from Loyola University Medical Center (LUMC) (IRB #215495) for this study. This is a retrospective review of the electronic medical record (Epic, Verona, WI) and Epic appointment statistics report (custom-built by author R.J.) from all scheduled in-person outpatient visits at a multi-physician, academic, general, and sub-specialty otolaryngology practice from January 2019 to December 2021. Data on patient demographics, clinic visit type, appointment adherence, and method of scheduling (in-person or over the phone vs. online self-scheduling) were collected. Age was stratified into three groups: younger patients (age under 18 years), adults (age 18-65 years), and older patients (over 65 years). Audiology-only, allergy-only, and patients scheduled to see a mid-level provider (nurse practitioner or physician assistant) were excluded. No-shows were classified as missed appointments without any notification to the clinic. Late cancellations were visits canceled within 24 hours of their scheduled appointment time. The formula for the no-show rate and nonattendance rate (which includes late cancellations) is as follows, with "#" representing the number of documented visits:

$$No\, show\, rate = \frac{\#\, of\, no\, shows}{\#\, of\, no\, shows + \#\, of\, completed\, visits}$$

$$Nonattendance\, rate = \frac{\#\, of\, no\, shows + \#\, of\, late\, cancellations}{\#\, of\, no\, shows + \#\, of\, late\, cancellations + \#\, of\, completed\, visits}$$

Data were collected for scheduled visits at two hospital-based and four satellite ambulatory clinics. The hospital-based clinics included the Loyola Outpatient Center and the Cardinal Bernardin Cancer Center at LUMC in Maywood, IL. Providers included three general otolaryngologists, five head and neck oncology surgeons, three pediatric otolaryngologists, one laryngologist, three rhinologists, five otologists, and one facial plastic and reconstructive surgeon. March 2020 was identified as the start of the nationwide lockdown for the COVID-19 pandemic in the United States. Visits scheduled prior to March 2020 were classified as pre-COVID-era visits, and visits scheduled after March 2020 were classified as COVID-19-era visits.

Statistical analysis

Data were compiled in Microsoft Excel 2018 (Microsoft Corporation, Redmond, Washington). Results of continuous variables are presented as means ± standard deviation (SD). The results of categorical variables are presented as frequencies (%). A two-tailed t-test was used to analyze differences between continuous variables, while the chi-square test was used to analyze differences between categorical variables. Logistic regression was used for multivariate analysis. All tests were two-sided at the 0.05 significance level. Analysis was conducted using IBM SPSS Statistics for Windows, Version 21 (Released 2012; IBM Corp., Armonk, New York).

## Results

**Figure 1:**
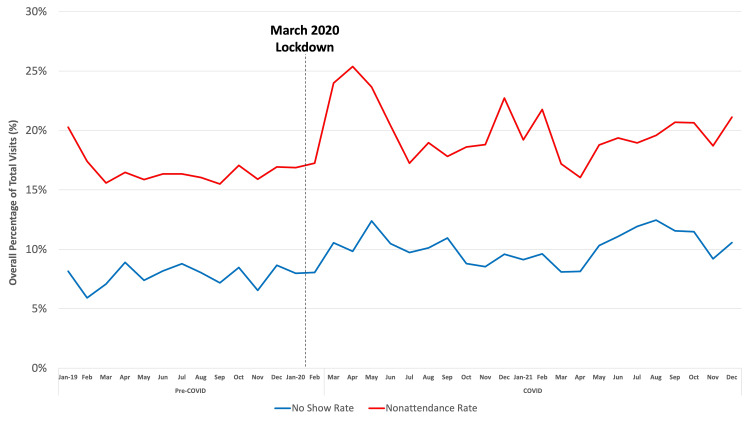
The overall rate of nonattendance plotted over time in months

Over a period of three years, a total of 121,347 clinic visits were scheduled by 37,883 patients. The average age of patients was 50.9 ± 23.4 years. Overall, 48.2% (n = 18,260) were male, and 51.8% (n = 19,623) were female. The overall no-show rate for all clinic visits was 8.9%, while the overall nonattendance rate was 18.3%. When analyzing individual patients, the total percentage of patients who no-showed was 2.1% (n = 796), while the total percentage of patients who nonattended (including late cancellations) was 5.3% (n = 2,008). Of those patients who no-showed, 6.34% (n = 50) no-showed more than once. Visits of patients who no-showed multiple times made up 1.3% (n = 115) of all no-show visits. A statistically significant increase in nonattendance was noted for COVID-era visits when compared with pre-COVID-era visits (19.8% vs. 16.8%, p < 0.001) (Figure 1).

**Figure 2:**
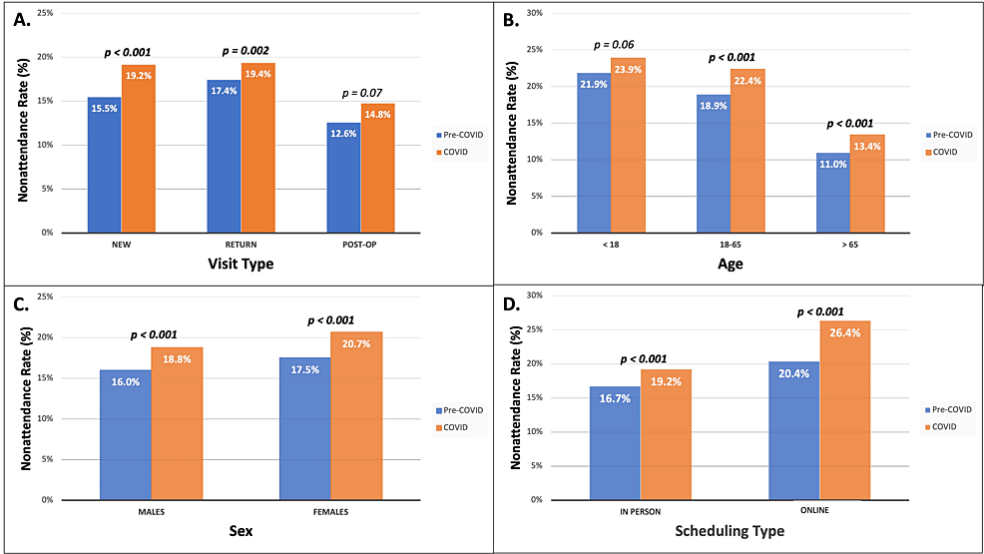
The rate of nonattendance by visit type, age in years, sex, and scheduling type

New, return, and postoperative visits comprised 39.7% (n = 48,175), 54.8% (n = 66,498), and 4.99% (n = 6,055) of all clinic visits (Figure 2A). When stratifying by visit type, the nonattendance rate for post-operative visits was significantly less (14%, p < 0.001) than for the other two groups: new and return (17.4%, 18.4%, respectively). There was no difference in the post-operative groups between the pre-COVID and COVID era (12.6% vs 14.8%, p = 0.07), although a significant difference existed for both new (15.5% vs 19.2%, p < 0.001) and return (17.4% vs 19.4%, p = 0.002) visits. Older patients (over 65 years) had a statistically significant decrease in the rate of nonattendance when compared with younger (under 18 years) (12.1% vs 22.7%, p < 0.001) and adults (18-65 years) (12.1% vs 20.7%, p = 0.02) (Figure 2B). A significant difference existed between the pre-COVID and COVID-era for adults (18.9% vs 22.4%, p < 0.001) and older patients (11.0% vs 13.4%, p < 0.001), while no such difference exists for younger patients (21.9% vs 23.9%, p = 0.06). Female nonattendance rate was significantly higher than that of males (19.1% vs 17.4%, p < 0.001), with both groups showing an increase in nonattendance rate in the COVID-era when compared to the pre-COVID era (females: 20.7% vs 17.5%, p < 0.001) (males: 18.8% vs 16.0%, p < 0.001) (Figure 2C). When comparing in-person/over-the-phone visits and online self-scheduling visits, the nonattendance rate was 17.9% and 25.3%, respectively (p < 0.001). Both groups saw a significant increase in nonattendance rate in the COVID-era when compared to the pre-COVID era (p < 0.001 in both groups) (Figure 2D).

**Figure 3:**
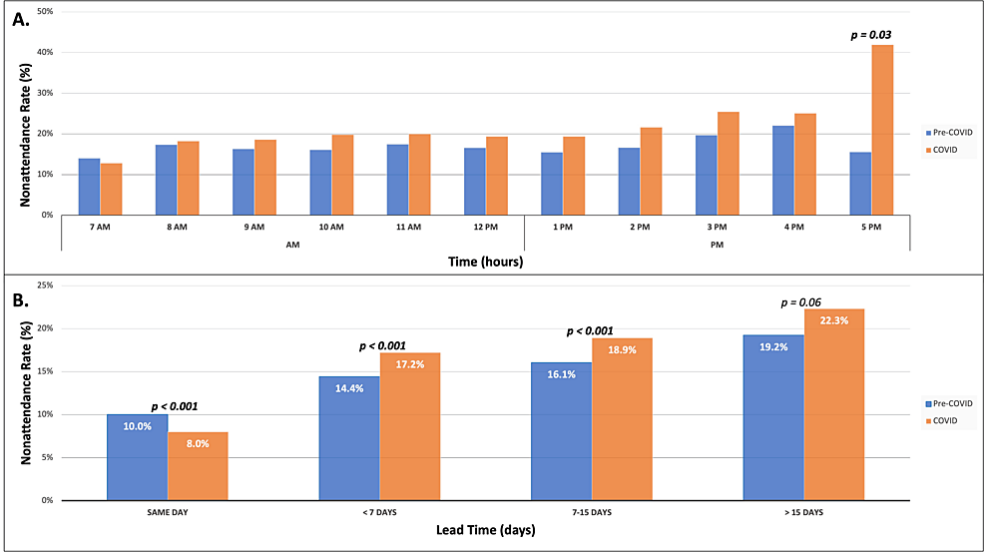
The rate of nonattendance by time of appointment and lead time

When stratifying by time of the appointment, there was a higher rate of overall nonattendance for afternoon (12 noon to 5 PM) appointments when compared to morning (7 AM to 12 noon) appointments (p = 0.04). Although the COVID pandemic did not appear to influence the nonattendance rate for morning (18.2% vs. 16.3%, p=0.16) and afternoon (26.7% vs. 17.9%, p=0.07) appointments, the nonattendance rate for the later timeslot of the day (5 PM) was significantly higher during the COVID-era (41.9% vs. 15.5%, p = 0.03) (Figure 3A). The association between lead time, defined as the number of days between the day of scheduling and the day of the visit, and the nonattendance rate is shown in Figure 3B. As lead time increased, the overall nonattendance rate was noted to increase as well (same day: 9.2%, within 7 days: 15.7%, 7-15 days: 17.6%, and greater than 15 days: 20.8%). The difference between pre-COVID-era nonattendance rates and COVID-era nonattendance rates by lead time was noted to be significant for same day (10.7% vs. 8.0%, p < 0.001), within 7 days (14.4% vs. 17.2%, p < 0.001), and 7-15 days (16.1% vs. 18.9%, p < 0.001). No such difference exists between pre-COVID and the COVID-era for patients who scheduled their appointment greater than 15 days out (19.2% vs. 22.3%, p = 0.06). Of note, the nonattendance rate for same-day visits declined during the COVID era when compared to the pre-COVID era, a statistically significant finding.

**Figure 4:**
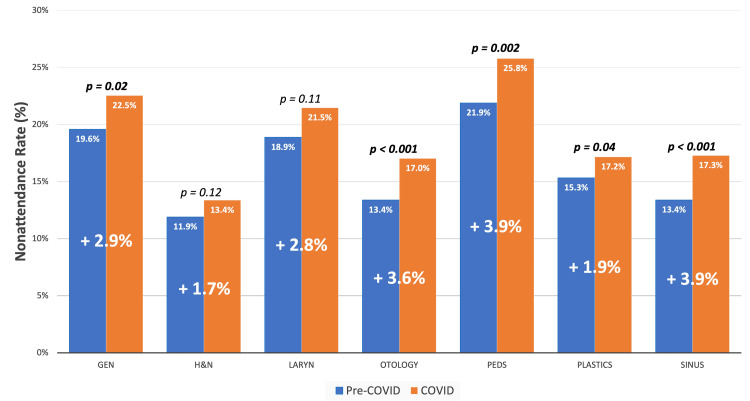
The impact of COVID-19 on the rate of nonattendance by otolaryngology subspecialty Gen, general; H&N, head and neck; Laryn, laryngology; Peds, pediatrics

The nonattendance rate for subspecialties within otolaryngology is shown in Figure 4. Overall, nonattendance for pediatric ENT clinics was the highest among all subspecialties (23.76%), as opposed to the nonattendance rate for head and neck cancer clinics (12.58%). When accounting for appointment length, a total of 5,820 hours of face-to-face patient interaction time was lost among all included otolaryngologists in this study. The average number of hours lost per provider per year is shown in Table 1. In this study, an average head and neck surgeon lost 34 face-to-face patient clinic hours compared to approximately 200 hours lost for the average pediatric otolaryngologist. The change in nonattendance rate in the pre-COVID era and COVID-era per subspecialty is also shown in Figure 4. Only for head and neck clinics and laryngology clinics was the difference between nonattendance rates for pre-COVID and COVID-era visits not significant. Again, head and neck clinics had the lowest increase overall in nonattendance rate in the pre-COVID era versus the COVID-era (+1.7%) when compared to pediatric (+3.9%) and sinus (+3.9%) clinics, which both had the highest increase in nonattendance rate across all subspecialties.

**Table 1:** Number of hours lost due to nonattendance Gen, general; H&N, head and neck; Peds, pediatrics

Specialty	Hours lost	Nonattendance per provider per year
Gen	1085	121
H&N	614	34
Laryngology	385	128
Otology	1464	122
Peds	1197	200
Plastics	450	75
Sinus	625	104

On multivariate regression, a significant association between nonattendance and younger age (p < 0.001), female gender (p=0.01), afternoon appointments (p< 0.001), and online self-scheduling (p< 0.001) was noted (Table 2). Age was noted to inversely correlate with nonattendance rate, while the strongest association existed between online self-scheduling and nonattendance rate. The association between lead time and rate of nonattendance was not significant (p=0.43).

**Table 2:** Multivariate logistic regression of factors associated with clinic nonattendance in otolaryngology patients CI, confidence interval

Factor	β	p	Odds ratio	95% lower CI	95% upper CI
Age	-0.012	<0.001	0.988	0.987	0.989
Sex	0.063	0.010	1.065	1.033	1.098
Lead days	0.010	0.430	1.001	1.000	1.001
Online self-scheduling	0.258	<0.001	1.295	1.219	1.375
Appointment time	0.000	<0.001	1.000	1.000	1.000

## Discussion

Clinic visit nonattendance represents a significant current healthcare challenge with widespread impact across all specialties of medicine. Often used as a metric for access to care, any strategy designed to reduce the rate of nonattendance remains an important topic of discussion. While many studies in the literature aim to identify patient and system factors associated with nonattendance in both academic and community settings, only a few studies have been identified within the field of otolaryngology [4-13]. Current national estimates suggest an overall 75% decline in outpatient otolaryngology visits at the beginning of the U.S. COVID-19 pandemic lockdown at the end of March 2020 [14]. This would be the first study to our knowledge evaluating the impact that the COVID-19 pandemic had on the rate of nonattendance in an academic otolaryngology practice.

Among the studies evaluating clinic visit nonattendance in otolaryngology, rates have shown to vary widely. One Canadian study reported a nonattendance rate of 24.4% for a multi-physician outpatient otolaryngology clinic in the context of a universal healthcare system. Factors associated with nonattendance were younger age (less than 39 years), males, and appointment day (Wednesdays) [13]. Another study at a multi-specialty academic otolaryngology practice at the University of Kentucky noted an overall no-show rate of 20% [4]. In contrast, one study of a single nonacademic single physician outpatient clinic reported a no-show rate of 8.4%, although the authors did not collect any additional patient factor data [5]. Miller et al. found that younger age, Black race, lower income, and Medicaid insurance were found to correlate with higher no-show rates in an academic practice [12]. Of note, their definition of “no-show” included patients who failed to show up to three or more of their scheduled appointments. Here, we classify no-shows as missed appointments without notification to the respective clinic. In order to capture a more accurate depiction of missed visits and their influence on individual clinics, our study included late cancellations (canceled visits within 24 hours of the scheduled appointment time) in our definition of “nonattendance,” which was the primary metric of this study. We observed an overall nonattendance rate of 18.3%, which is similar to other nonattendance rates reported in the literature for otolaryngology. The COVID-19 pandemic resulted in an overall increase in the rate of nonattendance by 3%, a significant difference from pre-pandemic rates. We also noted a significant association between nonattendance and younger age, female gender, afternoon appointments, and online self-scheduling.

Although the COVID-19 pandemic resulted in an increase in nonattendance for new and return patient visits, it did not appear to affect the rate of nonattendance for patients with postoperative visits. While the total number of elective surgeries may have decreased, patients' desire to follow up with their surgeon, to have their wound evaluated, or to receive pathology results did not appear to affect attendance in the COVID-19 era. As was the case in previous studies, our study found that age was negatively associated with the rate of nonattendance [4,6,13,15]. During the COVID-19 era, adults and older patients showed higher rates of nonattendance, while the rates in younger individuals (less than 18 years) did not change. Although older individuals generally may have poorer overall health and a greater need to seek medical assistance, fear of contracting COVID-19 may have encouraged older patients to stay indoors and not attend non-urgent appointments. Previous studies also noted an association between female gender and nonattendance, which was confirmed by the present study [4-6,13,15]. No difference in nonattendance was noted between male and female patients as a result of the COVID-19 pandemic. It is possible that due to the additional traditional responsibilities that society places on women with regard to home and family care, women may trend towards not attending non-urgent appointments, resulting in increased rates of nonattendance.

In this study, the rate of nonattendance for patients who self-scheduled their appointment online was 8% greater than the rate for patients who scheduled in-person or over-the-phone. This increased rate of nonattendance for online self-scheduling may be due to decreased perceived responsibility and the consequence of missing their visit. This mirrors current trends in the hospitality and food service industry, specifically with regard to “negligent diners” who make online restaurant reservations in advance but do not show up for their reservation [16].

There existed a higher rate of overall nonattendance for afternoon visits (12 PM to 5 PM). These hours may be less convenient for patients attending visits for non-urgent reasons. Interestingly, nonattendance rates in the COVID era increased solely for the 5 PM time slot, with upwards of more than 40% of all patients missing their appointments scheduled at the end of the day, compared to the 15% of patients who missed their appointments pre-pandemic. Our data suggested that nonattendance increased as lead time increased, with over 20% of patients missing their visit when scheduling more than two weeks in advance. This association did not appear significant on multivariate analysis. Of note, the nonattendance rate for same-day visits declined during the COVID era, compared to the pre-COVID era, from 10% to 8%, respectively. It is possible that those patients who requested same-day visits had more acute issues and were more willing to risk exposure to COVID-19.

Overall, nonattendance for pediatric otolaryngology clinics was the highest among all subspecialties, at 23.8%, but less than what is reported in the literature, with one study reporting an overall nonattendance rate of 33.0% [7]. In this study, head and neck clinics had lower reported rates of nonattendance, at 12.6%, and were least affected by the COVID-19 pandemic (+1.7%) when compared to pediatric (+3.9%) and sinus (+3.9%) clinics. Often, head and neck visits include new potential cancer patients or visits for cancer surveillance, with patients who have greater insight into their diagnoses and understanding of the importance of routine follow-up. The reasons for the high rate of nonattendance found in pediatric clinics are complex and multifaceted. Parents must weigh the benefits and costs of attendance for their child, which includes factors such as finding additional childcare and the financial implications of taking time off of work. For parents, the decision to attend or not may depend on their perception of the child's symptoms. A parent may choose not to attend if, for example, a child is symptomatic at the time of the pediatrician's referral but those symptoms resolve by the time they are scheduled to see the pediatric otolaryngologist.

Strategies and interventions for reducing nonattendance have been previously demonstrated in the literature. Among these are text-message appointment reminders, reducing the time between the scheduling of a visit and the visit itself, and "strategic scheduling" where patients are assigned to particular time slots based on their likelihood of missing their appointment [17-19]. This is in contrast to the popular practice of generic overbooking, which often causes issues with wait times and overtime for clinic staff. In the advent of the ever-increasing popularity of virtual applications and video conferencing, the average otolaryngologist, whether they agree or not, must recognize the impact that telemedicine will have on their practice moving forward. With the cessation of non-urgent visits and the imposition of stay-at-home orders early in the pandemic, telemedicine offers a way to provide care to patients remotely. An interesting study regarding telemedicine use in an academic otolaryngology department in New York City noted that physicians, overall, were less confident in making telemedical diagnoses due to a significant proportion (53% to 76%) of them reporting difficulty in performing a sufficient physical examination in at least 25% of teleconsultations [20]. Advances in the use of direct-to-consumer otoscopes for remote otoscopic examination and smartphones for oropharyngeal examination may help with this issue [21]. Overall, patients who trialed this "augmented outpatient otolaryngology teleconsultation" reported high rates of satisfaction, believed it facilitated earlier care, limited the cost and time of travel, and felt that their physician was able to perform a sufficient physical examination [20].

Our findings are from a tertiary academic center, and extrapolation to other medical specialties, private practices, or geographic regions may be limited. In addition, our study sample demographics may not reflect the patient population in other areas. Other socioeconomic factors, such as income status, level of education, and access to a computer or smartphone, may represent confounding variables that could influence the rate of nonattendance. Further research into more specific patient and system-wide factors, such as travel limitations or financial barriers and overall opportunity cost in the era of COVID-19, is needed. One important point to mention is that patients often experience no financial repercussions for their actions. Several industries, particularly the service industry, charge a fee for or otherwise hold people accountable for no-shows. Although some medical practices may collect a fee from patients for missed visits, the effect this has on the rate of nonattendance or on the financial loss from no-shows is uncertain, and further study is warranted.

Ultimately, the issue of nonattendance is multifactorial, and the utilization of system-wide or individual practice strategies (such as text-message appointment reminders or automated telephone reminders) to decrease nonattendance warrants further investigation. However, this study provides an important first look into the effect of COVID-19 on nonattendance rates, which can be used to direct further studies targeting clinical efficiency and identifying gaps in access to care.

## Conclusions

Both patient and administrative factors were found to impact rates of missed visits and late cancellations in this academic otolaryngology outpatient practice. In this study, young age, female gender, afternoon appointments, and online self-scheduling were associated with increased nonattendance. The COVID-19 pandemic significantly impacted nonattendance rates across all otolaryngologic subspecialties, with the largest impact on pediatric and sinus clinics and the lowest impact on head and neck clinics. This study provides an important first look into the effect of COVID-19 on clinic nonattendance rates, which can be used to direct further studies targeting improvements in timely and efficient care and identifying and addressing challenges in access to care.
